# PGPR-mediated enhancement of soil nutrients, rhizosphere microbial ecology, and plant growth: a review

**DOI:** 10.1038/s41522-026-00966-0

**Published:** 2026-03-23

**Authors:** Mengjiao Wang, Zhimin Xu

**Affiliations:** 1 School of Biological Science and Engineering, Hanzhong, China; 2 State Key Laboratory of Qinba Resource and Ecological Environment jointly established by the Ministry and Ministry of Education (Cultivation), Hanzhong, China; 3 Shaanxi Key Laboratory of Bioresources, Hanzhong, China; 4 Collaborative Innovation Center for Comprehensive Development of Biological Resources in Qinling-Ba Mountains, Shaanxi University of Technology, Hanzhong, China; 5 School of Nutrition and Food Sciences, Louisiana State University, Baton Rouge, LA USA

**Keywords:** Ecology, Ecology, Environmental sciences, Microbiology, Plant sciences

## Abstract

Plant Growth-Promoting Rhizobacteria (PGPR) are key bio-agents for sustainable agriculture. This review conceptualizes PGPR as rhizosphere engineers that enhance soil nutrients, restructure microbial networks, and boost plant stress tolerance. While their mechanisms are well-understood in the lab, a significant translational gap limits field efficacy due to inconsistent colonization and environmental context-dependency. We critically analyze this gap and propose integrated strategies—from advanced formulations to synthetic consortia—to unlock the reliable application of PGPR for global food security.

## Introduction

The escalated global demand for food production, coupled with gradually environmental degradation wrought by conventional agricultural practices has increased the search for sustainable and resilient farming systems. The historical understanding of beneficial plant-soil microbe interactions dates back over a century, with a pioneering work on nitrogen-fixing rhizobia in legume nodules^1^. The formal concept of “Plant Growth-Promoting Rhizobacteria” (PGPR) was first introduced by Joseph W. Kloepper in the late 1970s to describe a specific group of soil bacteria that, upon inoculation onto seeds or seedlings, could successfully colonize plant roots and exert beneficial effects on the host plant^2^. While the symbiotic relationship between *Rhizobium* and legumes served as an archetype, early PGPR research mainly focused on non-symbiotic, free-living bacteria, such as *Pseudomonas fluorescens* and *Bacillus subtilis*, noted for their potent biocontrol activities. Over time, the functional definition of PGPR has been expanded to include a wide range of bacterial genera from diverse phyla capable of improving plant growth through a multitude of mechanisms beyond biocontrol and nitrogen fixation with comprising nutrient solubilization, phytohormone production, and modulation of plant ethylene levels^3,4^.

Modern agriculture is confronted with profound challenges, according to the Food and Agriculture Organization. Widespread soil degradation affects approximately 33% of the world’s land area and result from erosion, salinization, compaction, and nutrient depletion. Concurrently, the overreliance on synthetic fertilizers is one of the major contributors to soil acidification, water eutrophication, and greenhouse gas emissions. Within this critical situation, PGPR have been rising as pivotal agents for a paradigm to shift traditional agriculture towards eco-intensified agriculture. By harnessing and optimizing beneficial plant-microbe interactions, PGPR offer a viable pathway to enhance soil fertility, facilitate efficient nutrient cycling, and bolster plant resilience against environmental stresses^5^. Their activities directly influence the soil’s physical structure, its chemical nutrient pools, and biological complexity of the microbial community, thereby creating a more robust and self-sustaining agricultural ecosystem^6,7^.

Therefore, this review aims to move beyond a mere cataloging of mechanisms. We offer two distinct contributions to the existing PGPR literature. First, we posit that the quintessential role of PGPR is that of a soil-plant system engineer—an agent that introduces targeted perturbations to reprogram the rhizosphere’s physicochemical and biological networks, leading to emergent system properties that enhance plant fitness and soil health. We first synthesize evidence across hierarchical levels, from molecular dialogs to ecosystem functions, to substantiate this integrative perspective. Second, and more critically, we confront the central paradox in PGPR research: the profound chasm between well-understood laboratory potential and inconsistent field efficacy. Unlike previous reviews that primarily catalog successful cases, we explicitly dissect the sources of this variability—poor rhizosphere competence, context-dependent regulation of beneficial traits, and formulation instability—and identify the research frontiers that must be bridged to transform PGPR from a promising concept into a reliable agricultural technology. By critically examining the roots of this translational gap, we identify the pivotal research frontiers that must be bridged to unlock the full promise of PGPR for sustainable agriculture and ecosystem restoration.

## Fundamental concepts of PGPR and the rhizosphere nexus

### Historical perspective, definition, and major taxonomic groups

The term “Plant Growth-Promoting Rhizobacteria” (PGPR) was introduced in the late 1970s to describe a specific group of soil bacteria existing upon inoculation onto seeds or seedlings, could successfully colonize plant roots and provide beneficial effects on the host plant^8^. While the symbiotic relationship between *Rhizobium* and legumes had long been established as the archetype for beneficial plant-microbe interactions, early PGPR research focused mainly on non-symbiotic, free-living bacteria noted for their potent biocontrol activities against soil-borne pathogens, such as *Pseudomonas* fluorescens and *Bacillus* subtilis^8^. Over time, the definition of PGPR expanded to include a wide range of bacterial genera from diverse phyla, which are capable of improving plant growth through a multitude of mechanisms that extend beyond biocontrol and nitrogen fixation. These encompass the solubilization of insoluble phosphates and potassium—a phenomenon first documented in the mid-twentieth century^9,10^—as well as the production of siderophores for iron chelation, a strategy recognized early in rhizosphere research^8^. The scope of PGPR action also includes the synthesis of phytohormones such as auxins, with the bacterial production of indole-3-acetic acid (IAA) being systematically detailed in earlier reviews^11^, and the production of the enzyme ACC deaminase, which modulates plant ethylene levels under stress and was formally described as a key mechanism by Glick et al.^12^.

This evolution in understanding has cemented the status of PGPR as indispensable components of sustainable agriculture for offering a biological means to reduce dependency on chemical fertilizers and pesticides and enhancing overall soil health and ecosystem resilience at the same time^7^. The major taxonomic groups of PGPR, their common ecological niches, and their primary mechanisms of action are diverse (Table 1).

**Table 1:** Major taxonomic groups of PGPR, their common habitats, and primary mechanisms

Bacterial genus	Common habitat	Primary PGPR mechanisms	Example species
*Pseudomonas*	Rhizosphere, root surfaces	P-solubilization, siderophores, antibiotics	*P. fluorescens, P. putida*
*Bacillus*	Soil, rhizosphere	P-solubilization, antibiosis, ISR	*B. subtilis, B. amyloliquefaciens*
*Rhizobium*	Legume root nodules	Nitrogen fixation	*R. leguminosarum*
*Azotobacter*	Rhizosphere, free-living	Nitrogen fixation	*A. chroococcum*
*Azospirillum*	Rhizosphere, associative	Nitrogen fixation, IAA production	*A. brasilense*
*Pantoea*	Phyllosphere, rhizosphere	IAA production	*P. agglomerans*
*Klebsiella*	Rhizosphere, endophytic	ACC deaminase, N-fixation	*K. pneumoniae*
*Burkholderia*	Soil, rhizosphere	Biocontrol, nutrient mobilization	*B. contaminans*

Notes: *ISR* induced systemic resistance, *IAA* indole-3-acetic acid

### The rhizosphere: a dynamic interface for plant-soil microbe dialog

The rhizosphere, the narrow zone of soil directly influenced by plant root exudates, constitutes one of the most dynamic and biologically active ecosystems on the Earth^13^. It is characterized by intense biochemical exchanges, primarily driven by a diverse cocktail of root-derived compounds including sugars (e.g., glucose, fructose), amino acids (e.g., glutamate, aspartate), organic acids (e.g., citric, malic, oxalic acids), phenolic compounds (e.g., flavonoids, tannins), fatty acids, and sterols^14^. These exudates function as both powerful chemical attractants and nutrient sources, selectively recruiting and sustaining specific microbial communities in the bulk soil. This establishes a sophisticated feedback loop wherein the plant, through its exudate profile, “manages” its associated microbiome. In return, the recruited microbes, including PGPR, significantly enhance plant nutrient acquisition, stress tolerance, and pathogen defense^15,16^. For instance, legumes secrete specific flavonoids (e.g., genistein, luteolin) to attract symbiotic rhizobia to initiate nodulation and nitrogen fixation. Tea plants exude phenolic acids that enrich beneficial *Pseudomonas* and *Bacillus* species^17^.

The successful establishment of PGPR within this competitive environment is a critical first step, requiring effective chemotaxis towards root signals, firm attachment to root surfaces, and the ability to outcompete indigenous microorganisms for resources and niches. Critically, this firm attachment often serves as the foundation for the development of complex multi-cellular biofilms, which act as a protected and coordinated microenvironment essential for long-term persistence and functional activity in the rhizosphere. PGPR employ various strategies for this competition, including resource competition for carbon and nitrogen, spatial competition for root adhesion sites, antibiosis through the production of antimicrobial compounds like phenazines and lipopeptides, and signal interference by disrupting the quorum-sensing systems of other microbes^18^.

Beyond initial chemotaxis and attachment, the formation of multicellular biofilms is a key strategy for PGPR to secure a niche in the rhizosphere. These biofilm structures, encased in self-produced extracellular polymeric substances (EPS), enhance bacterial tolerance to abiotic stresses (e.g., drought, pH fluctuation) and biotic threats (e.g., predation, competition). The biofilm mode of life facilitates intensive cell-to-cell communication via quorum sensing, which coordinates the collective expression of public goods, such as siderophores, antibiotics, and exoenzymes. Thus, the biofilm is not merely a physical aggregate but a functional consortium that underpins the very mechanisms—nutrient solubilization, pathogen inhibition, and stress resilience—through which PGPR promote plant health.

**Fig. 1:**
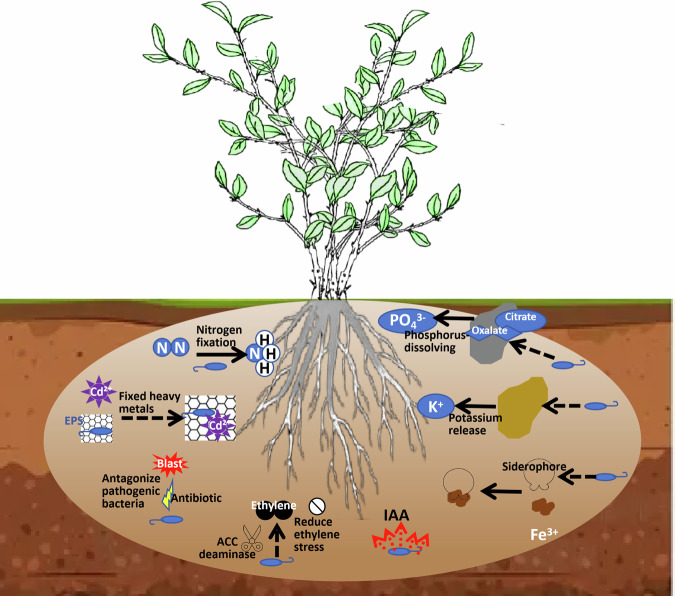
Multifunctional mechanisms of PGPR in the rhizosphere. This schematic illustrates the key processes mediated by plant growth-promoting rhizobacteria (PGPR) in the rhizosphere. These include the mobilization of macro- and micronutrients (e.g., nitrogen fixation, phosphate solubilization, siderophore production), the formation of biofilms on root surfaces for enhanced colonization, and the direct and indirect promotion of plant growth through phytohormone production (e.g., IAA) and stress alleviation.

This complex interplay of the signaling, competition, and cooperation makes the rhizosphere as a crucial nexus where plant-soil microbe partnerships are formed and functionally executed to ultimately influence plant health and agroecosystem productivity^13^. The multifunctional mechanisms through different PGPR operations in this complex environment are summarized in Fig. 1.

## PGPR-mediated enhancement of soil nutrients and synergies with amendments

PGPR instrumentally improve soil fertility by increasing the bioavailability of both essential macronutrients and micronutrients, thereby directly contributing to reduced application rates of synthetic fertilizers^19^. This section combines the mechanisms of nutrient mobilization and their synergistic interplay with organic inputs.

### Macro-nutrient mobilization

Nitrogen fixation: PGPR contribute to soil nitrogen through two main pathways: symbiotic and associative/free-living fixations^20^. Symbiotic fixers, such as *Rhizobium* and *Bradyrhizobium*, form specialized structures (nodules) on the roots of legume plants. Within these nodules, the bacteria convert atmospheric dinitrogen (N₂) into ammonia using the oxygen-sensitive nitrogenase enzyme complex. This process involves an intricate molecular dialog, which is initiated by flavonoid signals from the host root leading to bacterial nod factor secretion and eventual nodule organogenesis^21^. In contrast, associative nitrogen fixers like *Azospirillum brasilense* and free-living fixers like *Azotobacter chroococcum* colonize the rhizosphere or root surfaces of non-legumes (e.g., cereals) and fix nitrogen without forming specialized structures. These bacteria enhance nitrogen availability via biological nitrogen fixation (BNF) and reduce the dependence on synthetic N fertilizers^22^. Examples of nitrogen-fixing PGPR and their effects are provided in Table 2.

**Table 2:** Examples of nitrogen-fixing PGPR and their host plants

PGPR strain	Host plant	Location/soil type	Key methodological findings	Effect on plant/N soil	Reference
*Azospirillum brasilense*	Maize	Brazilian Cerrado soil	Inoculation via seed coating; measured by ^15^N isotope dilution	30% N derived from air (Ndfa)	^22^
*Pseudomonas stutzeri* A15	Rice	Paddy fields, China	Isolated from rice roots; nitrogenase activity measured by acetylene reduction assay	Reduced N fertilizer requirement by 30%	^67^
*Rhizobium leguminosarum*	Pea	Loamy soil, UK	Nodule occupancy and ^15^N tracing	80% plant N from fixation	^68^
*Flavobacterium* sp.	Rapeseed	Agricultural soil, China	Consortium (*Pseudomonas* + *Azotobacter*); NH_4_^+^ measurement	35–40% soil NH_4_^+^ increase	^45^
*Herbaspirillum seropedicae*	Rice	Flooded paddy, Philippines	GFP-tagged colonization assay; nifH gene expression	Significant acetylene reduction activity	^69^
*Azospirillum brasilense* Sp245	Wheat	Cerrado soil, Brazil	Inoculation via irrigation; ^15^N natural abundance	20–30% N derived from air (Ndfa)	^70^

Phosphate and potassium solubilization: Phosphorus is an abundant element in soil, but largely in insoluble forms and unavailable to plants. Phosphate-solubilizing bacteria (PSB), including key PGPR genera like *Pseudomonas* and *Bacillus*, address this limitation by secreting low-molecular-weight organic acids (e.g., gluconic, citric, oxalic). These acids chelate cations (such as Ca²⁺ in alkaline soils or Al³⁺ and Fe³⁺ in acidic soils) bound to phosphate (e.g., in tricalcium phosphate or rock phosphate) or protonate the insoluble mineral phosphates, thereby releasing plant-available orthophosphate ions (H₂PO₄⁻/HPO₄²⁻) into the soil solution^9,10,23,24^. Some PSB also produce the enzymes like phosphatases that mineralize organic phosphorus. The mechanisms and efficacies of representative PSB are summarized in Table 3.

**Table 3:** Mechanisms and efficacy of phosphate-solubilizing bacteria (PSB)

PGPR strain	Organic acid/enzyme	Experimental context (soil pH, P source)	Solubilization efficacy	Plant growth effect	Reference
*Bacillus megaterium*	Gluconic, Citric	Calcareous soil (pH 8.2), Tricalcium P	45% P solubilized in vitro	30% increase in P uptake in wheat	^71^
*Pseudomonas fluorescens*	Oxalic, Citric	Acidic soil (pH 5.5), Rock phosphate	52% soluble P released	25% root biomass increase in tomato	^23^
*Bacillus aryabhattai*	Gluconic	Metal-contaminated soil, Zn/Pb presence	48% P uptake increase in tea plant	Reduced Pb bioavailability	^40^
*Penicillium bilaiae*	Citric, Oxalic	Neutral prairie soil (Canada), rock phosphate	35% P mobilized in field	15% yield increase in canola	^72^
*Aspergillus niger*	Gluconic, Citric	Acidic lateritic soil (India), aluminum phosphate	High P solubilization in vitro	Improved P uptake in maize	^73^
*Rhizophagus irregularis* (AMF)	Phosphatase	Low-P agricultural soil (France)	Mineralization of organic P	Critical for P nutrition in multiple crops	^74^

Potassium release: Potassium (K) is the third major macronutrient, but a significant portion is trapped in silicate minerals like biotite and muscovite. Potassium-solubilizing bacteria (KSB), such as *Pseudomonas kribbensis* and *Bacillus mucilaginosus*, exude organic acids (e.g., oxalate, citrate) that weather these K-bearing minerals to release soluble K⁺ into the soil solution^25^. This process is particularly vital in degraded or K-deficient soils. The action of KSB is listed in Table 4.

**Table 4:** Potassium-solubilizing PGPR and their effects on plant growth

PGPR strain	Produced organic acid	Experimental context	Solubilization efficacy	Plant response	Reference
*Pseudomonas kribbensis*	Oxalate, Citrate	Organic paddy soil	40% K release from mica	25% increase in grain	^75^
*Bacillus mucilaginosus*	Citric, Oxalic	Pot trial, wheat	35% soluble K increase	Enhanced N uptake and biomass	^25^
*Burkholderia* sp.	Not specified	Machine oil-contaminated soil	Significant mineral weathering	Improved K availability	^76^
*Fraturia aurantia*	Tartaric, Citric	Mica, calcareous soil (Iran)	Significant pH reduction	20% increase in K uptake by wheat	^77^
*Bacillus edaphicus*	Not specified	Illite, saline soil	Increased exchangeable K	Improved growth of cotton under salinity	^78^

### Micro-nutrient availability enhancement

Beyond macronutrients, PGPR play a critical role in mitigating micronutrient deficiencies, particularly of iron (Fe). In aerobic and alkaline soils, iron predominantly exists in its insoluble ferric (Fe³⁺) form, rendering it largely unavailable to plants and leading to chlorosis. To overcome this, many PGPR synthesize and secrete siderophores, which are high-affinity iron-chelating compounds classified into catecholates, hydroxamates, and carboxylates^26^. These siderophores solubilize ferric iron by forming stable complexes that can be recognized by specific receptors on bacterial and plant root membranes for facilitating iron uptake^27^. This process not only alleviates iron chlorosis in plants but also confers an indirect competitive advantage to the PGPR by sequestering a vital nutrient and limiting its availability to pathogenic microbes. Furthermore, the metal-chelating property of siderophores extends to other metals, including heavy metals like zinc, lead, and cadmium, thereby reducing their phytotoxicity and contributing to plant metal stress tolerance^28^ (Table 5).

**Table 5:** Siderophore-producing PGPR in iron acquisition and metal detoxification

PGPR strain	Siderophore type	Host plant	Experimental context	Effect	Reference
*Pseudomonas* sp.	Pyoverdine	Cucumber	Alkaline soil, pH 8.3	Chlorosis reduction; 40% Fe uptake increase	^79^
*Bacillus subtilis*	Catecholate	Tea plant	Zn/Pb contaminated soil	35–50% less metal translocation	^40^
*Streptomyces* sp.	Hydroxamate	Wheat	Calcareous field	Improved Fe and Zn content in grains	^80^
*Pseudomonas putida* B10	Pyoverdine	Arabidopsis/Peach	Hydroponic, Fe-deficient	Rescued chlorosis; altered root architecture	^81^
*Streptomyces pilosus*	Desferrioxamine B	Calcareous soil (Spain)	Field trial with peanut	Increased chlorophyll content and pod yield	^82^

### Synergistic effects with organic amendments

The efficacy of PGPR is often significantly amplified when combined with organic amendments, creating a synergistic system that benefits both soil health and microbial function. This synergy operates through a biphasic mechanism that extends beyond simple carrier effects.

Organic amendments—such as spent mushroom substrate (SMS), biochar, compost, and vermicompost—provide a rich source of organic carbon, serving as a primary energy substrate for PGPR and the wider soil microbiome^29^. Their porous structure offers micro-niches that protect inoculant cells from predation, desiccation, and UV radiation, while their high water-holding capacity and buffering ability stabilize pH fluctuations^30^. When used as carriers, these materials enhance bacterial survival during inoculation and establishment, and can be engineered for controlled release (e.g., via alginate encapsulation or biochar adsorption), ensuring a prolonged rhizosphere presence^31^.

Following colonization, PGPR actively decompose the amendment matrix, releasing plant-available nutrients (N, P, K, micronutrients), humic substances, and disease-suppressive compounds (e.g., antibiotics, siderophores). This secondary metabolite pool further stimulates plant growth and indigenous beneficial microbes, creating a self-reinforcing fertility loop. Thus, the amendment transitions from a protective carrier to a functional output of PGPR activity.

The practical relevance of this synergy is illustrated across multiple cropping systems. In our own work with blueberry, co-application of SMS and PGPR increased soil organic matter by 18%, root biomass by 1.8-fold, and suppressed *Fusarium* pathogens, exemplifying circular economy principles through agricultural waste valorization^31^.

In rice paddies, co-inoculation of *Bacillus* and *Pseudomonas* with composted chicken manure and SMS boosted seedling vigor by 32–45% and significantly reduced sheath blight (*Rhizoctonia solani*) incidence, an effect attributed to both direct antagonism and enrichment of indigenous antagonistic taxa^32^.

In wheat, biochar-immobilized PGPR enhanced seedling biomass 1.6-fold compared to liquid inoculant alone; the porous biochar matrix protected cells from desiccation and provided slow-release niches^30^.

In vegetable production, vermicompost fortified with *Azospirillum* and phosphate-solubilizing bacteria increased marketable yield by 25–30% while reducing synthetic fertilizer inputs by 25%^33^.

Collectively, these examples demonstrate that organic amendments not only improve PGPR survival but also magnify their agronomic benefits across diverse environments. Representative synergistic combinations and their reported effects are summarized in Table 6.

**Table 6:** Synergistic effects of PGPR with organic amendments

Amendment type	PGPR strain(s)	Application method	Key findings	Reference
SMS	*Pseudomonas fluorescens*	Adsorption, 10⁸ CFU/g	1.8× root biomass; 18% SOM increase; *Fusarium* suppression	^31^
Biochar	*Bacillus aryabhattai*	Immobilization	Enhanced microbial network complexity; 30% available K increase	^30^
Compost	*Azospirillum* sp.	Seed coating	25% higher N retention; improved seedling vigor in maize	^83^
Vermicompost	*Pseudomonas corrugata*	Mixing with potting medium	Enhanced soil enzyme activities; suppressed Rhizoctonia in tomato	^33^
Dairy Manure	*Bacillus subtilis*	Co-composting	Accelerated composting, reduced pathogens, improved tomato seedling vigor	^84^

A critical yet underexplored dimension is the role of organic amendments as selective filters that shape the trajectory of PGPR-mediated microbiome engineering. Amendments with high C:N ratios (e.g., SMS, sawdust-based substrates) favor oligotrophic, cellulolytic microorganisms that may compete with copiotrophic PGPR inoculants. Conversely, nitrogen-rich amendments (e.g., manure, legume residues) create a copiotrophic environment that selects for r-strategist PGPR such as *Pseudomonas* and *Bacillus*. This selective pressure can either reinforce or counteract the intended microbiome shift, implying that amendment selection should be mechanistically matched to both the PGPR strain’s ecophysiology and the desired functional outcome—a principle seldom considered in current application studies. Future research must therefore integrate microbial ecology into formulation design, moving beyond empirical trial-and-error toward predictive, trait-based pairing of amendments and inoculants.

### Context dependency and limitations of PGPR-mediated nutrient mobilization

While the nutrient-mobilizing mechanisms of PGPR are well-established in vitro and under controlled conditions, their efficacy in field soils is far from guaranteed. The secretion of organic acids for phosphate solubilization, for instance, is often downregulated in the presence of readily available phosphorus or under carbon-limited conditions—a common scenario in bulk soils compared to the rhizosphere hotspot^34^. Similarly, nitrogen fixation by associative PGPR is highly sensitive to soil nitrogen status; elevated ammonium or nitrate typically represses nitrogenase activity, rendering the trait functionally inactive in fertilized agricultural systems. Furthermore, the expression of siderophore biosynthesis genes is tightly regulated by iron availability, and in iron-replete soils, this mechanism contributes little to plant nutrition. These context-dependent regulatory circuits explain why a strain that performs robustly in a pot trial may fail to enhance nutrient availability in a heterogeneous field environment. Acknowledging these limitations is not a dismissal of PGPR utility, but a prerequisite for designing inoculation strategies—such as using derepressed mutants or combining strains with complementary regulatory profiles—that can function predictably across diverse soil contexts.

## PGPR modulation of rhizosphere microbial ecology and function

The involvement of PGPR into the soil ecosystem actively reshapes the structural and functional dynamics of the resident rhizosphere microbiome and acts as a form of biological engineering^35^. This section details the modern techniques used to study these changes and impacts on the microbial community.

### Modern techniques for microbiome analysis

Understanding of these complex changes has been revolutionized by high-throughput molecular techniques. The 16S rRNA gene amplicon sequencing (e.g., of the V3–V4 hypervariable regions) provides detailed taxonomic profiling of bacterial and archaeal communities and reveals the shifts in diversity indices (alpha-diversity like Shannon and Chao1) and community composition differences (beta-diversity visualized via PCoA or NMDS)^36^. Internal Transcribed Spacer (ITS) sequencing gives analogous insights for fungal communities^37^. In addition to taxonomy, shotgun metagenomics sequences all the DNA in a sample, providing a comprehensive view of the functional gene potential of entire microbiome and identifying genes involved in nitrogen fixation (*nifH*), phosphate solubilization (*pqq*, *gcd*), siderophore production (pvd), and antibiotic synthesis (*phz*)^38^. Metatranscriptomics (RNA-seq) takes the step further by capturing the genes that are actively being expressed in situ, thereby distinguishing active functions from latent genetic potential. For instance, in tea rhizosphere studies, metatranscriptomics revealed the upregulation of *phoD* (alkaline phosphatase) and *acdS* (ACC deaminase) genes in PGPR-treated soils, which directly linked the microbial activity to phosphorus acquisition and stress ethylene mitigation^17^. Additionally, network co-occurrence analysis (e.g., SparCC, MENA) identifies keystone taxa and interprets microbial interaction patterns impacted by PGPR, which provides insights into community stability and resilience^39^.

### Structural shifts in microbial community composition

Inoculation with specific PGPR strains can induce significant and often predictable shifts in the taxonomic composition of rhizosphere microbial community. These shifts are highly context-dependent and vary by soil type, plant species, and management practices. For example, under conditions of heavy metal contamination, inoculation with metal-tolerant *Pseudomonas* or *Bacillus* strains can enrich the rhizosphere for other metal-resistant taxa from phyla, such as Proteobacteria (e.g., *Sphingomonas*, *Burkholderia*) and Chloroflexi, which often harbor genes for extracellular polymeric substance (EPS) biosynthesis and metal ion binding and facilitate metal immobilization with reducing phytotoxicity^40^. Similarly, the addition of nutrient-rich organic amendments like SMS alongside PGPR can create a selective environment that favors fast-growing (*r*-strategist) bacterial populations, such as Pseudomonadaceae and Bacillaceae, over slower-growing fungi, which potentially shift the dominant pathways of nutrient cycling within the soil food web towards a bacterially-dominated system. It enhances short-term nutrient turnover^32^. These PGPR-induced changes are documented across multiple studies as summarized in Table 7.

**Table 7:** Impact of PGPR inoculation on rhizosphere microbial community composition based on high-throughput sequencing studies

PGPR strain(s)	Host plant	Experimental context	Enriched taxa (Beneficial)	Suppressed taxa (Pathogens/Negative)	Method Used	Reference
*Pseudomonas fluorescens*	Tea	Zn/Pb contaminated soil	*Sphingomonas*, *Chloroflexi*, *Bradyrhizobium*	*Fusarium*, *Ascomycota* (pathogenic)	16S rRNA/ITS sequencing	^40^
*Bacillus subtilis* consortium	Blueberry	SMS-amended acidic soil	*Actinobacteria*, *Bacillaceae*, *Lysobacter*	*Pythium*, *Fusarium oxysporum*	Metagenomics	^31^
*Azospirillum brasilense*	Maize	Nitrogen-deficient field	*Rhizobiales*, *Burkholderiaceae*, *Nitrosospira*	*Fusarium* spp.	16S and ITS2 sequencing	^22^
Mixed PGPR	Rice	Paddy soil, salinity stress	*Halomonas*, *Bacteroidetes*, *Rhodobacteraceae*	*Pseudomonas syringae*, *Fusarium graminearum*	16S rRNA sequencing	^57^
*Bacillus amyloliquefaciens* FZB42	Arabidopsis	Gnotobiotic system	Root colonization induces systemic resistance	Alters root exudation profile	Metagenomics & Transcriptomics	^1^
*Rhizobium* sp.	Common bean	Drought stress	Enriches for Actinobacteria	Shifts fungal community	ITS sequencing	^85^

### Enhancement of microbial network complexity and stability

Beyond simple taxonomic profiles, co-occurrence network analysis has revealed that PGPR can enhance the architectural complexity and stability of microbial interactions. Inoculation often results in microbial networks with higher connectivity, greater modularity, and increased robustness^41^. These changes suggest that a more resilient microbial community is better buffered against environmental perturbations. Keystone taxa hold a disproportionately large influence on network structure and often emerge or are strengthened following PGPR application. For instance, in tea rhizospheres, PGPR inoculation resulted in more complex networks where *Cercozoa* (a group of protozoan predators) emerged as keystone taxa^17^. These protozoa regulate bacterial population dynamics through top-down predation and contribute to organic matter turnover and suppress pathogen outbreaks, thereby promoting ecosystem stability. Additionally, PGPR that express ACC deaminase (e.g., *Pseudomonas migulae*) lower the stress ethylene levels in plant roots, which helps preserve microbial niche structure and prevents the collapse of mutualistic interactions during drought or flooding and further contribute to network stability^42^.

### Functional activation of soil enzyme activities

The structural reorganization of the microbiome driven by PGPR translates into tangible enhancements of microbial functionality, most readily observed through the stimulation of soil enzyme activities^43^. PGPR consistently upregulate the activity of key enzymes involved in the biogeochemical cycling of nutrients. This includes urease and protease, which are critical for nitrogen mineralization and release of ammonium and phosphatases (both acid and alkaline), which catalyze the mineralization of organic phosphorus into plant-available phosphate and β-glucosidase and cellulase, which drive carbon cycling by breaking down cellulose and other carbohydrates to facilitate soil organic matter decomposition^44^.

In blueberry cultivation trials, inoculation with PGPR strains such as *Bacillus* and *Pseudomonas* significantly increased acid phosphatase activity by 2.1–2.5 times. The enhancement directly correlated with improved root phosphorus absorption efficiency and increased plant biomass^45^. This enzymatic activation is particularly pronounced in soils amended with organic materials like SMS, where PGPR utilize the additional carbon sources to boost their own metabolic activity and enzyme production. These functional shifts underscore the role of PGPR as effective bio-catalysts that activate the inherent nutrient cycling services in the rhizosphere, thereby reducing dependence on external fertilizers and enhancing the soil innate fertility.

### The paradox of microbiome engineering: perturbation versus resilience

The observation that PGPR inoculation can restructure the rhizosphere microbiome is often implicitly framed as a beneficial outcome. However, this framing warrants critical scrutiny. While shifts toward greater network complexity and enzyme activity are desirable, PGPR-induced community restructuring does not always translate to improved plant outcomes, nor is it universally stable. In some cases, the introduced strain fails to establish a detectable niche, and the resident microbiome exhibits strong homeostatic resilience, rapidly returning to its pre-inoculation composition^46^. In others, successful invasion by the PGPR may displace functionally redundant but locally adapted taxa, inadvertently reducing the community’s functional redundancy and long-term resilience to novel disturbances. Moreover, the current literature—including our own studies—overwhelmingly reports short-term taxonomic shifts (days to weeks), whereas the ecological legacy of PGPR inoculation over multiple growing seasons remains severely underexplored. Whether repeated inoculation builds sustainable, self-amplifying benefits or leads to inoculant dependency and microbiome fatigue is an open question that future research must address.

## PGPR-driven plant growth promotion and stress alleviation: integrated mechanisms

The cumulative benefits of PGPR-mediated improvements in soil nutrition and microbiology ultimately manifest as enhanced plant growth, development, and resilience to a wide array of stresses. This section integrates direct hormonal effects with stress alleviation mechanisms.

### Phytohormone-mediated growth promotion

A direct and potent mechanism of plant growth promotion is the bacterial synthesis and modulation of phytohormones. A large proportion of PGPR are capable of producing auxins, most notably indole-3-acetic acid (IAA). Bacterial IAA synergizes with endogenous plant auxin to profoundly influence root architecture and stimulate the formation of lateral roots and root hairs^3^. This morphological expansion significantly increases the root surface area and enhances the plant’s capacity to explore a larger soil volume for water and nutrients. For example, in tea and blueberry production systems, inoculation with IAA-producing PGPR like *Pantoea agglomerans* was shown to increase lateral root density by 30–50% and root biomass by up to 40%^45,47^.

Other PGPR strains produce cytokinins or gibberellins, which can promote cell division, delay senescence, and stimulate shoot elongation^48^. Furthermore, the enzyme ACC deaminase, produced by bacteria such as some *Pseudomonas putida* and *Variovorax* species, cleaves the immediate ethylene precursor (1-aminocyclopropane-1-carboxylate, ACC) in the plant rhizosphere. By reducing the root’s ACC pool, these bacteria lower the potential for stress-induced ethylene synthesis^49^. This reduction in ethylene prevents the typical growth inhibition associated with various abiotic stresses, such as drought, flooding, salinity, and heavy metal toxicity, and allows plants to maintain growth under adverse conditions. For instance, *Pseudomonas putida* inoculation led to a 35% higher survival rate for blueberries in cadmium-contaminated soil^45^.

### Abiotic stress alleviation

PGPR are highly effective in priming plants for enhancing tolerance to abiotic stresses. Under heavy metal stress, PGPR employ a dual strategy. Firstly, they facilitate the extracellular immobilization of metals through the secretion of exopolysaccharides (EPS) and siderophores, which chelate metal ions and reduce their bioavailability and uptake by plant roots^50^. Secondly, and perhaps more crucially, PGPR prime the plant intrinsic defense systems. Inoculation with PGPR is consistently shown to upregulate the plant antioxidant machinery, leading to a significant increase in the activity of enzymes like superoxide dismutase (SOD), catalase (CAT), and peroxidase (POD)^51,52^. This enhanced enzymatic scavenging capacity efficiently neutralizes reactive oxygen species (ROS) generated under stress and protects cellular components, particularly the photosynthetic apparatus, from oxidative damage. For example, inoculation with Bacillus licheniformis in tea plants under Zn/Pb stress resulted in a 2.8-fold increase in SOD activity and significantly reduced oxidative leaf damage^40^. Similar protective effects were observed under drought and salinity, where PGPR-inoculated plants often exhibit better osmotic adjustment through the accumulation of proline and soluble sugars, and maintain higher leaf water potential and photosynthetic rates^53,54^. Examples of PGPR involved in phytohormone production and stress alleviation are provided in Table 8.

**Table 8:** Examples of PGPR in phytohormone production and plant stress alleviation

PGPR strain	Host plant	Phytohormone/Enzyme	Mechanism of action	Effect on plant growth and stress alleviation	Reference
*Pantoea agglomerans*	Tea	IAA (33.5 mg/L)	Stimulates lateral root proliferation	40% increase in root biomass; Enhanced drought tolerance	^47^
*Pseudomonas putida*	Blueberry	ACC deaminase	Lowers ethylene levels under metal stress	35% higher survival rate in Cd-contaminated soil	^25^
*Bacillus subtilis*	Rapeseed	Cytokinins	Promotes cell division and delays senescence	20% increase in seed yield under nutrient deficiency	
*Bacillus licheniformis*	Tea	- (SOD/CAT induction)	Activates antioxidant defense system	2.8x SOD activity; Reduced oxidative leaf damage	^40^
*Achromobacter piechaudii*	Tomato	ACC deaminase, ABA modulation	Reduces stress ethylene, improves water relations	Enhanced growth under drought and salinity	^86^
*Pseudomonas fluorescens*	Chickpea	Gibberellin production	Direct GA biosynthesis, alters root architecture	Increases nodulation and yield under salinity	^87^
*Burkholderia phytofirmans* PsJN	Grapevine	Multiple (IAA, ACCd)	Priming of stress-responsive genes	Confers cold and heat tolerance	^88^

### Integrated mechanisms and cross-talk in PGPR functionality

While the direct and indirect mechanisms of PGPR are often studied individually, their true power lies in their integrated and synergistic functioning within the rhizosphere ecosystem. A critical yet underexplored area is the cross-talk between these mechanisms, where one process directly influences or enhances another. For instance, the secretion of organic acids (e.g., gluconic, oxalic) for phosphate solubilization concurrently acidifies the rhizosphere^55^. This local pH reduction can significantly alter the bioavailability of heavy metals (e.g., Cd, Pb) and micronutrients (e.g., Fe, Zn), thereby reshaping the microbial community and influencing plant uptake patterns^51^. Similarly, the production of indole-3-acetic acid (IAA) often works in concert with ACC deaminase activity^56^. Under stress conditions, IAA promotes root proliferation and expands the surface area for nutrient and water uptake, while ACC deaminase cleaves the ethylene precursor ACC and mitigate stress-induced growth inhibition^57^. This dual action creates a synergistic loop where plant growth is promoted simultaneously with stress resilience. However, it is equally important to recognize that our understanding of this cross-talk remains fragmentary, and its outcomes are not universally synergistic. Under certain conditions, mechanism interference can occur. For example, high levels of bacterial IAA can induce excessive ethylene production in plant roots—a stress response that may overwhelm the capacity of ACC deaminase to cleave its precursor, paradoxically exacerbating growth inhibition^58^. Similarly, the organic acids exuded for phosphate solubilization can, in acidic soils, further lower rhizosphere pH to levels that mobilize phytotoxic aluminum, negating any nutritional benefit. These potential trade-offs are rarely examined in single-strain, single-mechanism studies. Future research must move beyond isolating synergistic anecdotes and systematically map the environmental and regulatory boundaries within which these mechanisms operate harmoniously versus antagonistically.

## Synergistic applications in contaminated soil remediation and future perspectives

**Fig. 2:**
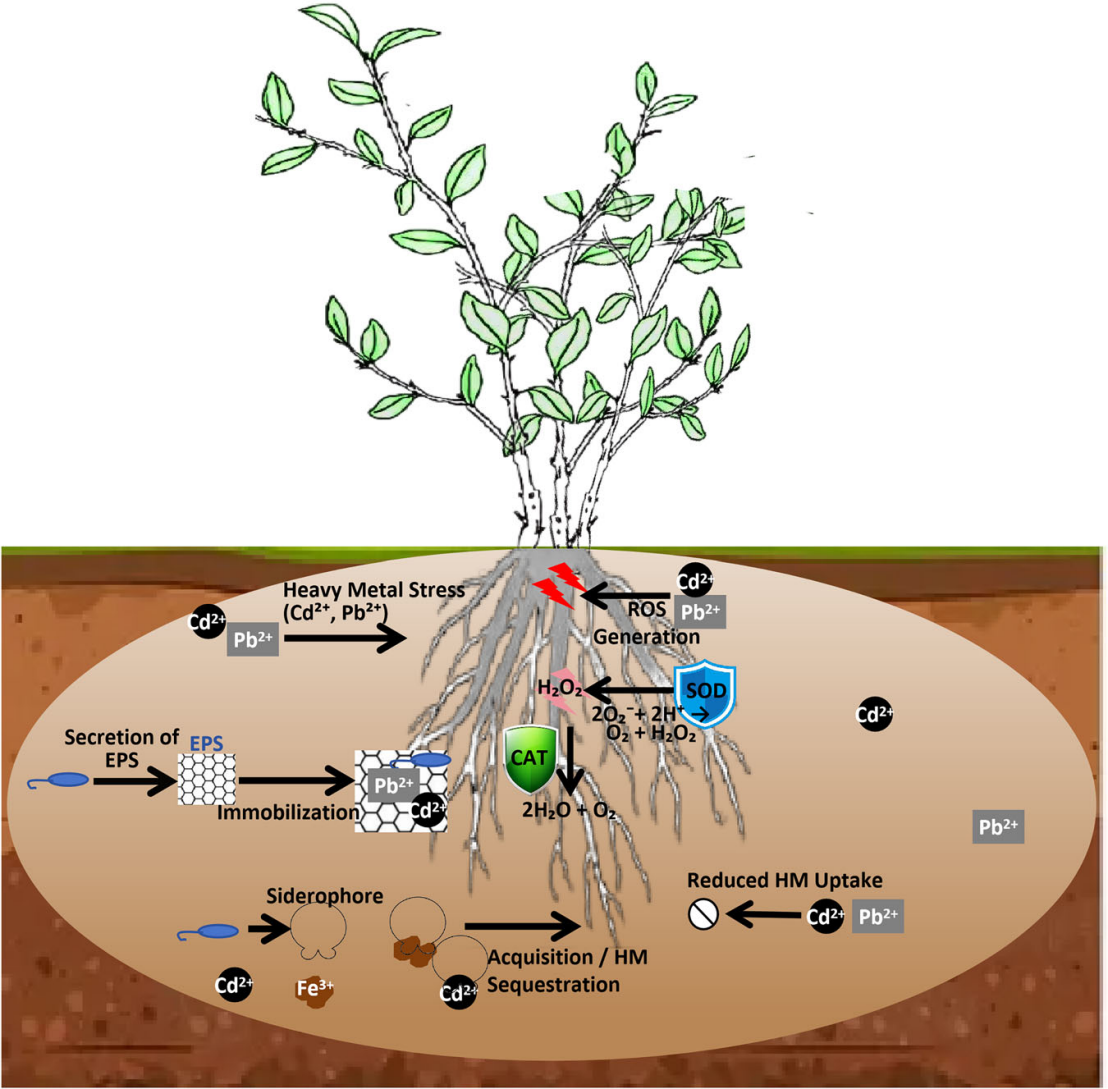
Dual mechanisms of PGPR in alleviating heavy metal stress and enhancing phytoremediation. PGPR facilitate plant survival in contaminated soils through two complementary strategies. (1) Extracellular immobilization: Secretion of exopolysaccharides (EPS) and siderophores chelates metal ions in the rhizosphere, reducing their bioavailability and phytotoxicity. (2) Intracellular defense priming: PGPR upregulate the plant’s antioxidant enzyme system (e.g., SOD, CAT, POD), which scavenges reactive oxygen species (ROS) and protects cellular structures from oxidative damage.

The combination of PGPR with plants is an approach known as microbe-assisted phytoremediation and offers a highly promising and ecologically sustainable strategy for the rehabilitation of soils contaminated with heavy metals and organic pollutants^59^. PGPR enhances the efficiency of phytoremediation through several interconnected mechanisms, as illustrated in Fig. 2.

### Synergistic applications in contaminated soil remediation

PGPR enhance phytoremediation efficiency by increasing metal bioavailability through rhizosphere acidification and secretion of organic acids and siderophores. PGPR can solubilize metal precipitates and increase their bioavailability for plant uptake in phytoextraction strategies^60^. Conversely, some PGPR can sequester metals through biosorption to their cell walls and exopolymeric substances (EPS) to reduce metal mobility and leaching of phytotoxicity for stabilizing the contaminants in the root zone (phytostabilization)^61^. PGPR also induce plant synthesis of metal-chelating compounds (e.g., glutathione, phytochelatins) and enhance the antioxidant system by mitigating oxidative damage and improving plant health and survival in contaminated soils^62^. By improving plant nutrition and reducing stress, PGPR promote greater root and shoot biomass, which directly increases the total metal extraction capacity (for phytoextraction) or enhances the stability of the root zone (for phytostabilization)^63^.

In our studies, *Bacillus* and *Pseudomonas* strains isolated from tea rhizospheres significantly enhanced glutathione synthesis in *Camellia sinensis* thereby reducing Pb and Cd translocation to shoots by 35–50% and demonstrating a phytostabilization effect^40^. Field applications, however, must contend with significant challenges, which are primarily the inconsistent survival and metabolic activity of the introduced PGPR inoculants over time. For instance, in a blueberry field trial, the population of inoculated *Pseudomonas fluorescens* declined by approximately 40% after 12 months and highlighted the critical need for ongoing research into advanced carrier materials, such as SMS-biochar composites and encapsulation technologies, to improve the shelf-life, persistence^31^. Case studies of PGPR-assisted phytoremediation are presented in Table 9.

**Table 9:** Case studies of PGPR-assisted phytoremediation of heavy metal contaminated soils

Metal(s)	Plant species	PGPR strain	Location/soil type	Remediation efficiency and key findings	Reference
Cd, Pb	*Camellia sinensis* (Tea)	*Bacillus* sp. TE3, *Pseudomonas* sp. TP1	Fujian, China; Acidic red soil	50% reduction in shoot Cd; 48% increase in root Pb immobilization	^40^
Zn, Cu	*Zea mays* (Maize)	*Azospirillum brasilense*	Smelter-impacted area	30% higher metal accumulation in roots; 25% increase in biomass	^89^
As	*Pteris vittata* (Hyperaccumulator)	*Arthrobacter* sp.	Contaminated paddy soil	40% greater As extraction in fronds; Enhanced glutathione levels	^90^
Cd	*Brassica juncea*	*Variovorax* sp.	Pot experiment; Loam soil	35% higher Cd uptake in roots; Reduced chlorophyll degradation	^31^
Ni	*Alyssum murale*	*Microbacterium* sp.	Serpentine soil (France)	Increased Ni concentration in shoots by 30%	^91^
Zn, Cd	*Sedum alfredii*	*Burkholderia cepacia*	Mine tailings (China)	Enhanced metal extraction, increased biomass	^92^

### Future perspectives and research gaps

**Fig. 3:**
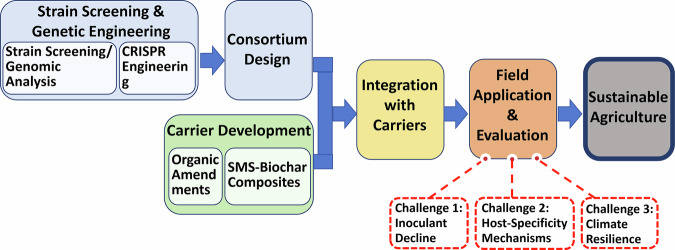
Roadmap for PGPR application and major challenges in field translation. This figure outlines the critical stages for transitioning PGPR from laboratory concepts to reliable field applications. It highlights the major bottlenecks—including inconsistent colonization, competition with native microbiomes, and formulation instability—that contribute to the “translational gap.” Potential solutions, such as advanced carrier formulations, synthetic microbial consortia (SynComs), and genetic engineering, are positioned as key interventions to bridge this divide.

While the promise of PGPR is immense, a persistent translational gap remains between laboratory efficacy and consistent field-scale performance. Addressing this gap requires a concerted focus on several key frontiers, as outlined in Fig. 3. A critical manifestation of this gap is the decline in inoculant viability over time—for example, a 40% reduction in *Pseudomonas fluorescens* population 12 months after field application^31^. Overcoming this barrier is not merely a formulation problem; it requires a multi-level strategy that integrates materials science, microbial ecology, and genetic engineering. Potential solutions can be grouped into three complementary intervention levels, which are summarized and compared in Table 10.

At the formulation level (immediate term), next-generation carrier technologies must move beyond simple adsorption. Encapsulation in alginate, chitosan, or polymer-biochar composites creates a protected microenvironment that shields cells from desiccation, UV radiation, and predation while enabling controlled release^64^. Double encapsulation (e.g., an alginate core with a chitosan coating) further extends shelf-life by adding an extra diffusion barrier. Fluidized bed drying and spray drying with protective osmoprotectants (trehalose, skim milk) can produce shelf-stable powders with >6 months viability at ambient temperatures—a critical requirement for scalability in low-resource agricultural systems. Emerging nano-formulations using mesoporous silica nanoparticles or nanoclay composites can deliver PGPR directly to root surfaces with unprecedented precision, though cost currently limits field-scale adoption.

Complementary to formulation advances, the ecological design of Synthetic Microbial Consortia (SynComs) offers a medium-term strategy to enhance persistence and functionality. Rather than deploying a single ‘superbug’ expected to perform all functions and outcompete all competitors, rationally designed SynComs distribute labor across multiple strains with complementary ecological strategies. Three principles derived from natural microbiome assembly are particularly relevant: functional redundancy—multiple taxa capable of the same function (e.g., phosphate solubilization) buffer against the loss of any single strain; metabolic cross-feeding—one strain’s waste product serves as a carbon or energy source for another, increasing community stability and resistance to invasion^65^; and spatial niche partitioning—combining rhizoplane colonizers, endophytic strains, and rhizosphere soil inhabitants minimizes direct competition while maximizing functional coverage of the root system. These principles remain dramatically underutilized in commercial inoculant development; translating them into practice will require high-throughput community assembly assays and predictive ecological models.

Over the longer term, strain improvement through genetic engineering can directly address intrinsic limitations of PGPR. CRISPR-based genome editing offers unprecedented opportunities to enhance rhizosphere persistence and activity. Targeted modifications could: delete negative regulators of biofilm formation (e.g., c-di-GMP phosphodiesterases) to lock strains into a high-adhesion phenotypic state; introduce biosynthetic pathways for antifungal metabolites or siderophores to directly suppress competitors; engineer stringent response mutants that maintain metabolic activity under carbon-limited conditions; and enhance osmoprotectant biosynthesis for improved drought tolerance^66^. Crucially, such modifications must be coupled with containment strategies (e.g., kill switches, auxotrophy) to address regulatory and public acceptance concerns associated with the environmental release of engineered strains.

Importantly, no single intervention will suffice. The 40% decline observed in our field trial^31^, likely arose from the convergence of multiple stressors—carbon limitation, predation, pH fluctuation, and competition. Therefore, stacking complementary solutions (e.g., encapsulating a SynCom of two engineered strains in a tailored organic carrier) will probably be necessary to achieve the >12-month persistence required for perennial crops and low-input systems. Future research must systematically evaluate such stacked interventions under realistic field conditions and across diverse agro-ecological zones. The strategies outlined above, along with their respective mechanisms, advantages, limitations, and technology readiness levels, are synthesized in Table 10 to guide researchers and product developers in prioritizing efforts to bridge the translational gap.

**Table 10:** Strategies to overcome PGPR inoculant instability and poor field persistence: mechanisms, advantages, limitations, and readiness levels

Strategy	Mechanism/Approach	Key advantages	Limitations/Challenges	Technology readiness level
Encapsulation (alginate, chitosan, polymer-biochar)	Cells immobilized in hydrated polymer matrix; controlled release	Protects from desiccation, predation, UV; extends shelf-life	Scale-up cost; viability loss during drying	TRL 4–6
Double encapsulation	Core-shell structure (e.g., alginate + chitosan)	Enhanced diffusion barrier; longer persistence	Complex manufacturing	TRL 3–4
Nano-formulations	Mesoporous silica, nanoclay, nano-emulsions	Precision delivery; root-targeted	High cost; regulatory uncertainty	TRL 2–3
Osmoprotectant formulation	Trehalose, skim milk, PEG in spray/fluidized bed drying	Ambient-temperature stability; low cost	Protection efficiency varies by strain	TRL 5–6
Synthetic Microbial Consortia (SynComs)	Functional redundancy, metabolic cross-feeding, spatial niche partitioning	Resilience to strain loss; multifunctionality	Design rules immature; inconsistent assembly	TRL 2–3
CRISPR-based strain engineering	Knockout of biofilm suppressors; introduction of competitive traits	Direct genetic control; heritable	GMO regulation; public acceptance	TRL 2–3
Stringent response engineering	Maintain activity under carbon limitation	Prolonged metabolic function	Fitness costs; off-target effects	TRL 1–2

## Conclusion

This review has synthesized evidence supporting the perspective of Plant Growth-Promoting Rhizobacteria (PGPR) as integral soil-plant system engineers. Rather than acting as mere providers of discrete nutrients or stress relievers, PGPR function by reprogramming the rhizosphere system—altering its physicochemical niche, restructuring microbial community networks, and modulating plant physiology—to generate emergent properties that enhance overall system resilience and productivity.

This systems-level understanding, however, starkly contrasts with the persistent translational gap observed in field applications, where efficacy is often inconsistent and context-dependent. Bridging this gap is the paramount challenge for realizing the full potential of PGPR.

Doing so requires not only technological innovation but also a conceptual shift in how the scientific community evaluates and positions PGPR. The narrative of PGPR as a standalone, plug-and-play bio-input must give way to a more nuanced framework that explicitly acknowledges their context dependency and integrates them as one component—rather than a substitute—within broader agroecosystem management.

The future of PGPR applications hinges on interdisciplinary research that converges microbial ecology, molecular biology, material science, and agronomy. By moving beyond proof-of-concept studies toward mechanistic dissection of failure modes—and by designing formulations, consortia, and deployment strategies explicitly to overcome these failure points—the field can unlock the potential of PGPR. Not as a universal panacea, but as a strategically deployable tool for building systemic resilience in agriculture.

## Data Availability

No datasets were generated or analyzed during the current study.
